# Supplementary figures and images for: Associations between academic achievement and weight status in a multi-ethnic sample of New Caledonian adolescents
Source: PLoS One. 2024 Oct 3;19(10):e0309782. doi: 10.1371/journal.pone.0309782 (PMC11449337; doi:10.1371/journal.pone.0309782)

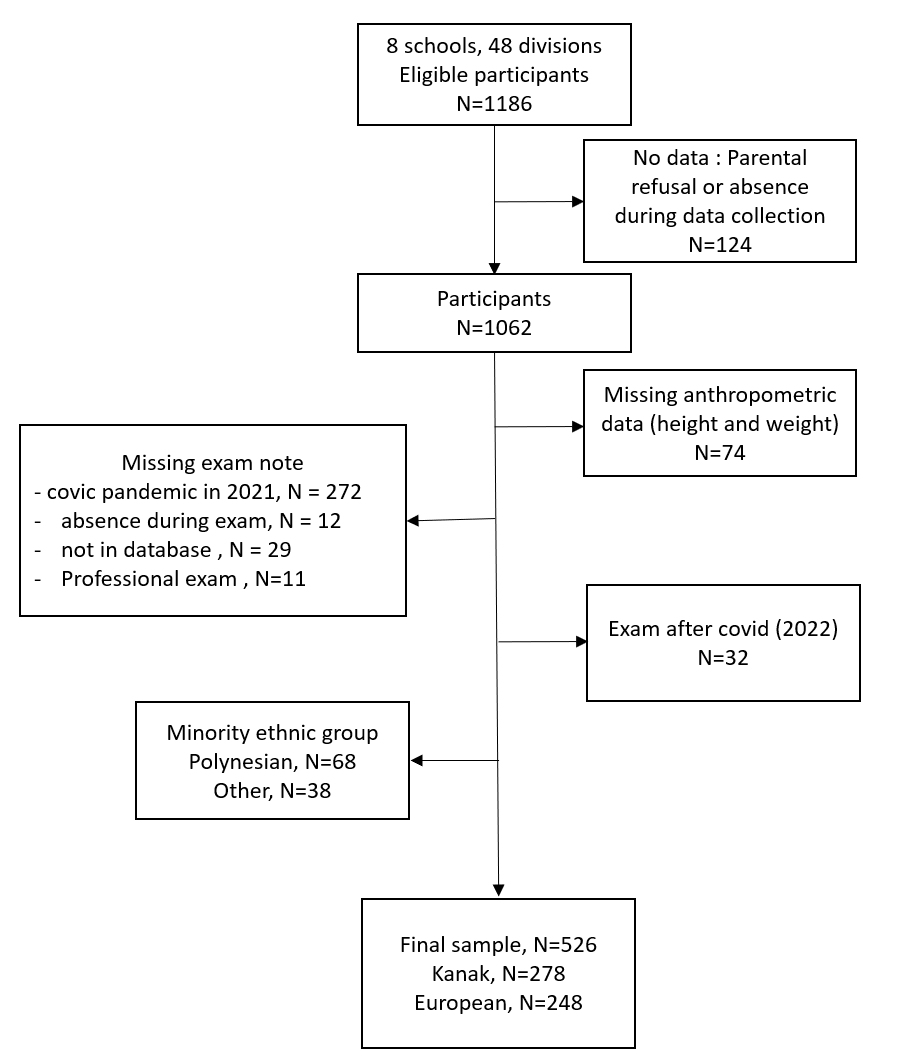

Supplement: S1 Fig — (TIF) [file pone.0309782.s001.tif]

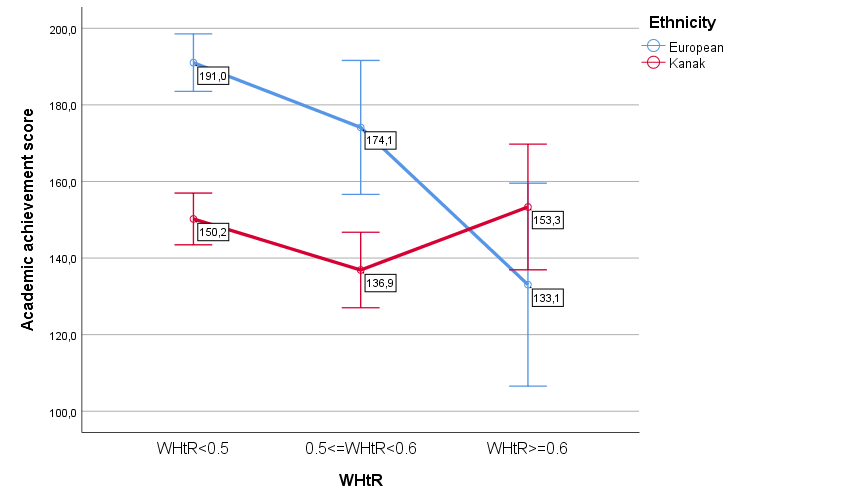

Supplement: S2 Fig — (TIF) [file pone.0309782.s002.tif]
