# Supplementary material for: Associations between academic achievement and weight status in a multi-ethnic sample of New Caledonian adolescents
Source: PLoS One. 2024 Oct 3;19(10):e0309782. doi: 10.1371/journal.pone.0309782 (PMC11449337; doi:10.1371/journal.pone.0309782)
Supplement: S1 Table — (DOCX) [file pone.0309782.s003.docx]

**Table S1: Hierarchical linear regression models examining predictors of academic achievement in European and Kanak adolescents.**

|  | **European**  **(*n* = 248)** | | | | | | **Kanak**  **(*n* = 278)** | | | | | |
| --- | --- | --- | --- | --- | --- | --- | --- | --- | --- | --- | --- | --- |
| **Step 1** |  | | | | | | | | | | | |
| ***R*² (Adj. *R*²)** | .25 (.23) | | | | | | .12 (.10) | | | | | |
| ***ΔF*** | 13.61 (*p* < .001) | | | | | | 5.89 (*p* < .001) | | | | | |
| **Variable** | ***B*** | ***SE*** | **β** | ***t*** | ***p*** | ***B*** | | ***SE*** | **β** | ***t*** | ***p*** |  |
| **SRI** | **-1.24** | **.33** | **-.222** | **-3.75** | **< .001** | -0.07 | | 0.24 | -0.018 | -0.30 | .761 |  |
| **Girls**† | 7.69 | 6.27 | .071 | 1.23 | .221 | **13.07** | | **5.18** | **0.146** | **2.53** | **.012** |  |
| **High SES**£ | **27.98** | **7.29** | **.258** | **3.84** | **< .001** | **22.98** | | **7.42** | **0.180** | **3.10** | **.002** |  |
| **Mid SES**£ | 17.86 | 9.44 | .125 | 1.89 | .060 | 16.27 | | 8.52 | 0.112 | 1.91 | .057 |  |
| **2019 exam#** | **52.52** | **7.84** | **.480** | **6.70** | **<.001** | **-22.48** | | **6.63** | **-.254** | **-3.53** | **<.001** |  |
| **2020 exam#** | **30.11** | **8.18** | **.264** | **3.68** | **<.001** | **-28.76** | | **7.19** | **-.289** | **-4.00** | **<.001** |  |
| **Step 2** |  | | | | | | | | | | | |
| ***R*² (Adj. *R*²)** | .27 (.25) | | | | | | .12 (.10) | | | | | |
| ***DF*** | 6.96 (*p* = .009) | | | | | | 2.09 (*p* = .149) | | | | | |
|  | ***B*** | **SE** | **β** | ***t*** | ***p*** | ***B*** | | SE | **β** | ***t*** | ***p*** |  |
| **WHtR** | **-132.79** | **50.35** | **-0.154** | **-2.64** | **.009** | -52.64 | | 36.41 | -0.085 | -1.45 | .149 |  |
| **SRI** | **-1.19** | **0.33** | **-0.213** | **-3.65** | **<.001** | -0.07 | | 0.24 | -0.018 | -0.30 | .761 |  |
| **Girls**† | 7.80 | 6.19 | 0.072 | 1.26 | .209 | **14.22** | | **5.23** | **0.159** | **2.72** | **.007** |  |
| **High SES**£ | **21.96** | **7.56** | **0.202** | **2.91** | **.004** | **23.08** | | **7.40** | **0.181** | **3.12** | **.002** |  |
| **Mid SES**£ | 11.65 | 9.61 | 0.082 | 1.21 | .227 | 16.04 | | 8.51 | 0.111 | 1.89 | .060 |  |
| **2019 exam#** | **51.81** | **7.75** | **.474** | **3.68** | **<.001** | **-23.47** | | **6.38** | **-.265** | **-3.68** | **<.001** |  |
| **2020 exam#** | **29.76** | **8.08** | **.261** | **-2.64** | **<.001** | **-27.99** | | **7.20** | **-.281** | **-3.89** | **<.001** |  |

Abbreviations: SRI: school remoteness index; SES, socioeconomic status; WHtR, waist to height ratio

Bold text indicates a statistically significant difference with a p-value less than .05.

Reference is: † Boys; £ Low SES; #2018 exam
