# Supplementary material for: Associations between academic achievement and weight status in a multi-ethnic sample of New Caledonian adolescents
Source: PLoS One. 2024 Oct 3;19(10):e0309782. doi: 10.1371/journal.pone.0309782 (PMC11449337; doi:10.1371/journal.pone.0309782)
Supplement: Attachment — Submitted filename: response to reviewer.docx [file pone.0309782.s004.docx]

**Associations between academic achievement and weight status in a multi-ethnic sample of New Caledonian adolescents**

**RESPONSES TO ACADEMIC EDITOR AND REVIEWERS**

We thank the reviewers and editors for their appraisals. We have done our best to address the concerns and hope that the manuscript is now suitable for publication. Please note that all the change has been tracked in red.

**Journal Requirements:**

**1. Please ensure that your manuscript meets PLOS ONE's style requirements, including those for file naming.**

This has now been done, as requested.

**2. We noticed you have some minor occurrence of overlapping text with the following previous publication(s), which needs to be addressed:**

We checked and rewrote the sentences concerned

**3. Please state what role the funders took in the study.**

This has been done.

**4. Please review your reference list to ensure that it is complete and correct.**

This has been verified.

**Reviewer #1:**

1)Abstract:
**In methodology kindly mention the study duration.**

This has now been done, as requested.

2) Introduction:

**The authors first can start with global burden of obesity and current trend with increase in obesity among adolescents.
To restrict introduction to 3-4 paragraphs.**

Thank you for this suggestion. We have revised the opening paragraph to include some information about the global burden of obesity, as requested. However, we have struggled to comply with the second request, namely to substantive shorten the Introduction. In our view, all the information currently presented in the Introduction is necessary to provide a full and rounded review of the literature, as well as to provide a clear justification for the present work. As such, while we have made minor edits to shorten the Introduction, we have not made any substantive revisions in this regard.

3) Methodology:
**Kindly mention the study design.**

This has been done, please see line 107.

**More detail description of participant selection have to be provided like inclusion criteria, total number of secondary schools and why only 8 eight schools were selected.**

All the inclusion criteria and the rational to selected eight schools has been described in the manuscript. See lines 123-134.

**What are exactly divisions.**

We apologise for any confusion. We now use the term “grade” throughout, which should hopefully be more familiar to readers.

**The authors have mentioned that study period commenced from 2018-2019, I would like to know why authors are commenting regarding 2020 and 2021 year. As it is understood the data collection period does not include these years.**

We apologise for any lack of clarity. As specified in the manuscript lines 151-152, participants took academic tests at the end of the ninth grade in 2018, 2019, or 2020 depending on their school grade during the data collection process (7th, 8th, or 9th year in 2018 or 2019).

**The authors have to mention who were measuring the anthropometric measurements in research team, are they are trained for measuring required anthropometric measurements?**

This has been done Please line 160.

4) Conclusion:

**Kindly include concluding points on European and Kanak ethnic groups.**

Thank you for this suggestion. In the final paragraph of the Discussion, we now briefly discussion implications of our findings.

Reviewer #2: Dear Author, The paper has significant findings.However, some clarifications are needed

Comments:
**1. in the introduction, what is the nutritional status of students in New Caledonia**

We apologise, but it is unclear what further information the reviewer is requesting. If the reviewer is requesting information about nutritional intake vs. nutritional demands of adolescents in New Caledonia, we are sorry to say that such data are not currently available.

**2. the sample size calculation was not stated**

This has been done Please line 117-122.

**3. study locations: how many schools are in urban and rural?**

This has been specified, please see line 123-129.

**4. Table 3 should be in scientific table**

We apologise that this was not done earlier. All tables have now been formatted according to the journal’s guidelines.

**5. How do you imply the results into practice?**

Thank you for this suggestion. In the final paragraph of the Discussion, we now briefly discussion implications of our findings.
